# Retraction Note: Human cathelicidin LL-37 enhance the antibiofilm effect of EGCG on Streptococcus mutans

**DOI:** 10.1186/s12903-017-0392-3

**Published:** 2017-06-26

**Authors:** Yi-jie Guo, Bo Zhang, Xue-song Feng, Hui-xun Ren, Ji-ru Xu

**Affiliations:** 1 Department of Pathogenic Microbiology and Immunology, School of Basic Medical Sciences, Xi’an Jiaotong University, Yanta West Road No.76, Xi’an, 710061 ShaanXi China; 2 Clinical Laboratory, AnKang City Central Hospital, Jinzhou South Road No.85, AnKang, 725000 ShaanXi China; 3 Key Laboratory of Environment and Genes Related to Diseases, Ministry of Education of China, Xi’an Jiaotong University, Yanta West Road No.76, Xi’an, 710061 ShaanXi China

## Retraction

The authors are retracting this article [1] because they do not have ownership of the data they report. A formal investigation by Tohoku University has concluded that the data reported in this article are the sole property of Tohoku University and have been reported in Bai et al. [2]. All authors agree with this retraction.

## References

[1] Guo Y-J, Zhang B, Feng X-S, Hui-xun Ren H-X, Xu J-R (2016). Human cathelicidin LL-37 enhance the antibiofilm effect of EGCG on Streptococcus mutans. BMC Oral Health.

[2] Bai L, Takagi S, Ando T, Yoneyama H, Ito K, Mizugai H, Isogai E (2016). Antimicrobial activity of tea catechin against canine oral bacteria and the functional mechanisms. J. Vet. Med..

